# Molecular characterization of Nipah virus from *Pteropus hypomelanus* in Southern Thailand

**DOI:** 10.1186/s12985-016-0510-x

**Published:** 2016-03-25

**Authors:** Supaporn Wacharapluesadee, Panumas Samseeneam, Mana Phermpool, Thongchai Kaewpom, Apaporn Rodpan, Pattarapol Maneeorn, Phimchanok Srongmongkol, Budsabong Kanchanasaka, Thiravat Hemachudha

**Affiliations:** World Health Organization Collaborating Centre for Research and Training on Viral Zoonoses, King Chulalongkorn Memorial Hospital, Faculty of Medicine, Chulalongkorn University, Bangkok, Thailand; Department of National Parks, Wildlife and Plant Conservation, Bangkok, Thailand

**Keywords:** Nipah virus, Bats, Strain, Transmission, Southern, Thailand

## Abstract

**Background:**

Nipah virus (NiV) first emerged in Malaysia in 1998, with two bat species (*Pteropus hypomelanus* and *P. vampyrus*) as the putative natural reservoirs. In 2002, NiV IgG antibodies were detected in these species from Thailand, but viral RNA could not be detected for strain characterization. Two strains of NiV (Malaysia and Bangladesh) have been found in *P. lylei* in central Thailand, although Bangladesh strain, the causative strain for the outbreak in Bangladesh since 2001, was dominant. To understand the diversity of NiV in Thailand, this study identified NiV strain, using molecular characterizations, from *P. hypomelanus* in southern Thailand.

**Findings:**

Pooled bat urine specimens were collected from plastic sheet underneath bat roosts in April 2010, and then monthly from December 2010 to May 2011 at an island in southern Thailand. Five in 184 specimens were positive for NiV, using duplex nested RT-PCR assay on partial nucleocapsid fragment (357 bp). Whole sequences of nucleocapsid gene from four bats were characterized. All 5 partial fragments and 4 whole nucleocapsid genes formed a monophyletic with NiV-MY.

**Conclusions:**

Our study showed that *P. hypomelanus* in southern Thailand and from Malaysia, a bordering country, harbored similar NiV. This finding indicates that NiV is not limited to central Thailand or *P. lylei* species, and it may be a source of inter-species transmission. This indicates a higher potential for a widespread NiV outbreak in Thailand. NiV surveillance in Pteropus bats, the major natural reservoirs, should be conducted continuously in countries or regions with high susceptibility to outbreaks.

## Background

Nipah virus (NiV) has been genetically characterized as Malaysia (NiV-MY) or Bangladesh (NiV-BD) strains, termed based on the country of their first outbreak, with fruit bats as the main reservoir [1]. Based on currently available complete N gene sequences, NiV-MY was obtained from Malaysia (*Pteropus hypomelanus*, *P. vampyrus*, sick pigs and patients) and Cambodia (*P. lylei*), while NiV-BD was obtained from patients from Bangladesh and India. Outbreak in Malaysia of NiV-MY in 1998 contributed to encephalitis with 39 % fatality rate [2]. Recent encephalitis outbreak (most likely caused by NiV) in Southern Philippines (2014) originated from fruit bats, transmitted to horses and then to humans, which was attributable to either horse slaughter or horse meat consumption [3]. Secondary man-to-man spread was evident, with acute encephalitis, severe influenza-like illness, or meningitis (82 % fatality). NiV-BD has been associated with the Indian (2001) and Bangladeshi (since 2001) outbreaks of encephalitis and respiratory distress syndrome, with a fatality rate of over 70 % [4]. Infectivity and pathogenicity of the two strains may be different, as NiV-BD seems to be associated with higher incidence of respiratory disease. A study found higher level of oral shedding in ferrets infected with NiV-BD than with NiV-MY [5]. However, both strains caused similar respiratory tract lesions in Syrian hamsters [6]. On the other hand, disease does not develop in Pteropid bats, whether infected naturally or experimentally [7–9].

Serological studies have demonstrated evidences of NiV infection in multiple bat species, including frugivorous and insectivorous bats. However, viral isolation and molecular characterization was mostly only successful in *Pteropus* species [10]. NiV-MY was isolated from urine samples of *P. hypomelanus* [7] and *P. vampyrus* [11] from Malaysia. NiV-MY has also been found in other *Pteropus* bats from various countries; *P. lylei* in Cambodia, nucleocapsid (N) sequence of 1599 bp, shared 98 % identity with the Malaysian patient (AF212302) [12] and *P. vampyrus* in Indonesia, matrix gene of 251 bp had 100 % identity to NiV-MY in the Malaysian patient (AF212302) and 99.6 % homology with the Malaysian *P. vampyrus* bat (FN869553) [13]. *P. giganteus* is believed to be the source of NiV infection in India and Bangladesh via contaminated date palm sap. NiV-BD has been detected from a liver homogenate of *P. giganteus* captured in India with its partial N gene (205 bp), showing 100 % homology with NiV infected patients in India (FJ513078.1) and Bangladesh (AY988601) [14]. On the other hand NiV was detected in *P. lylei* from Thailand, with sequences (357 bp) sharing 98–99 % nucleotide homology with Bangladeshi patients (AY988601) [15], but there has been no report of an outbreak in humans or other animals.

The intensive surveillance of NiV in *P. lylei* in central Thailand has been conducted yearly since 2002. To date, NiV RNA was only detected in *P. lylei* among two other Pteropus species in Thailand. Fifty two (88.1 %) NiV-BD and 7 (11.9 %) NiV-MY were reported between 2002 and 2008 [15–17]. NiV IgG antibody has been found in *P. hypomelanus* and *P. vampyrus* from southern Thailand, but NiV RNA could not be successfully detected [16]. The objective of this study was to identify and characterize the strains of NiV found in *P. hypomelanus*, in southern Thailand.

## Methods

**Fig. 1:**
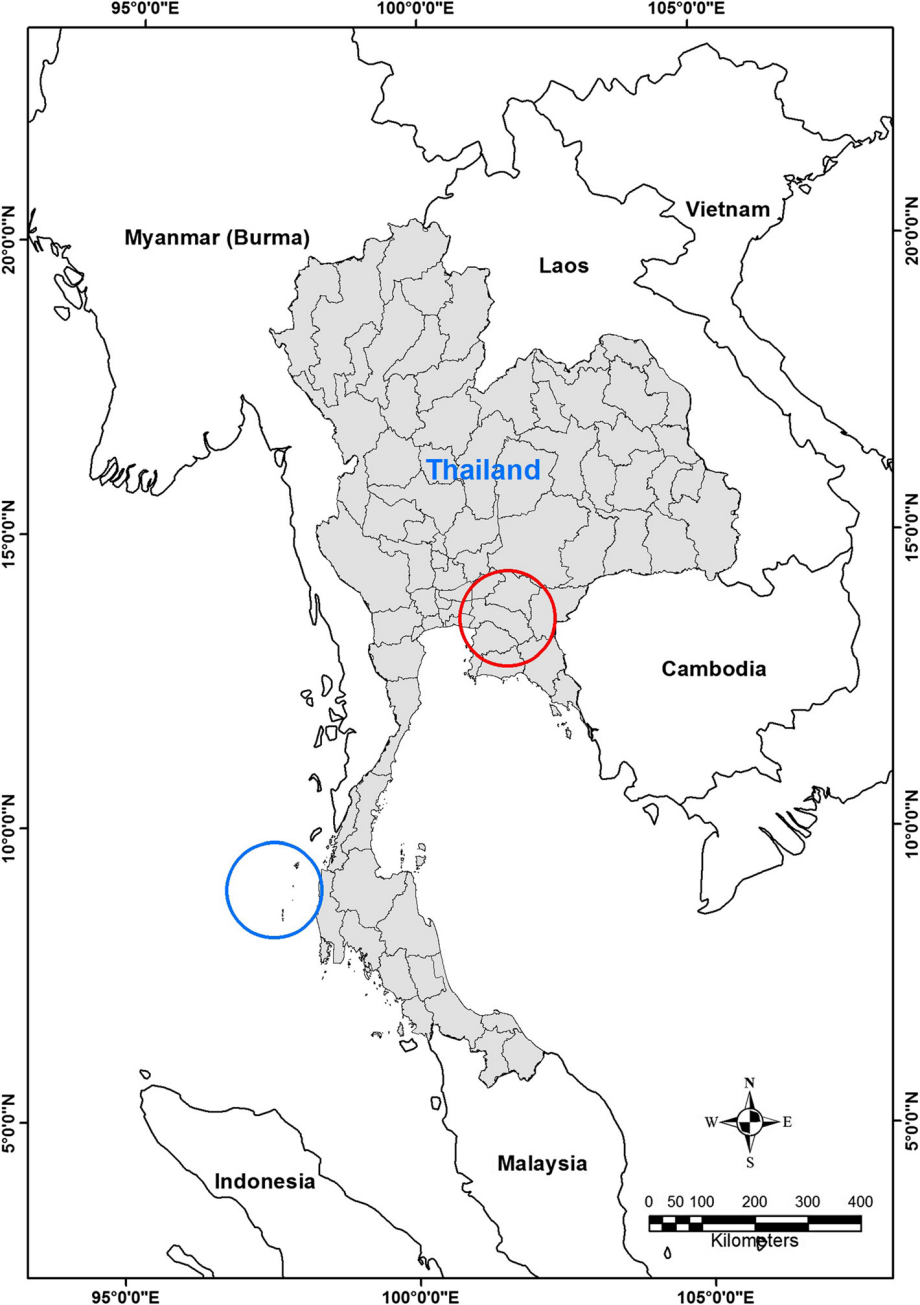
Locations in Thailand where bat urines specimens were collected from *P. hypomelanus* at the south (blue circle) and *P. lylei* at central (red circle)

**Fig. 2:**
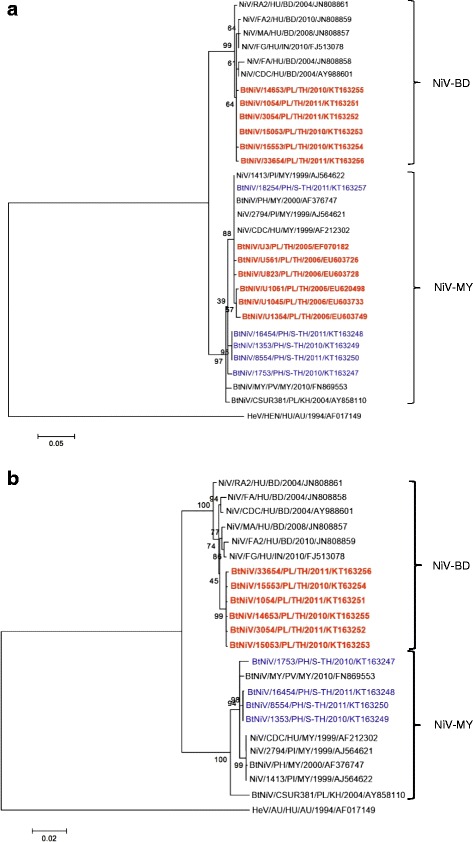
Phylogenetic trees of Nipah virus (NiV) gene; **a** partial coding sequence (cds) of nucleocapsid (N) gene (357 bp), and **b** complete cds of N gene (1599 bp). Maximum-likelihood method was used to analyze phylogenetics, using sequences available in GenBank. NiV-MY partial N gene from *P. lylei* [15] and additional whole N gene sequences of NiV-BD obtained in 2010–2011 from urine of *P. lylei* bats in Thailand (GenBank accession numbers KT163251- KT163256) were included. Alignments were constructed by Multiple Alignment Fast Fourier Transform (MAFFT). Bootstrap values were determined using 1000 replicates via MEGA 6.06. The tree was then visualized using FigTree program, version 1.4.2. GenBank accession numbers are shown for corresponding sequences. NiVs found in *P. hypomelanus* and *P. lylei* from Thailand are colored in blue and red respectively. Hendra virus (HeV) was used as the outgroup. Bt, Bat; HU, Human; PI, Pig; PV, *Pteropus vampyrus*; PH, *P. hypomelanus*; PL, *P. lylei*; MY, Malaysia, BD, Bangladesh; IN, India; KH, Cambodia; TH, Thailand; S-TH, Southern Thailand

Survey location was an island in Southern Thailand, a National Park, where only *P. hypomelanus* bats roosted in trees along the beach (Fig. 1). Numbers of bat population varied each month due to rain storms (Table 1). Bat urine samples were collected under roost trees with permission from the Department of National Parks, Wildlife and Plant Conservation (No. 0907.1/20713). The samples were collected once in April 2010, and then monthly from December 2010 to May 2011, using a plastic sheet protocol as previously described [18]. Two swabs of pooled bat urine collected from plastic sheet were stored in Lysis buffer (bioMérieux), transported on ice to the laboratory within 48 h and stored at −80 °C until analysis. The nucleic acid was extracted from bat urine using the NucliSENS easyMAG® extraction kit (bioMérieux). NiV RNA was first screened by duplex nested RT-PCR as previously described [17]. Only PCR positive specimens were further characterized for whole N gene by 3 additional PCR assays. All PCR positive specimens were directly sequenced on hemi-nested PCR products using an automated ABI PRISM 377 model sequencer. The sequence segments were assembled by BioEdit program [19]. Phylogenetic trees were generated by using maximum-likelihood method based on 357 bp (Fig. 2a) and 1599 bp (Fig. 2b) of N gene.

**Table 1:** PCR testing of urine specimens for Nipah virus (partial fragment, 357 bp), categorized by month and year of collection, from a single colony of *Pteropus hypomelanus* from an island in southern Thailand

Collected Dates	Population numbers^a^	No. of positive/total^b^ (%)	Identity similarity to AF376747^c^ (%)
			(GenBank number: identity/total nucleotides)
April 2010	800	2/28 (7.1)	KT163247: 1584/1599 (99.1 %)
			KT163249: 1589/1599 (99.4 %)
December 2010	228	0/7 (0)	-
January 2011	218	0/30 (0)	-
February 2011	675	1/24 (4.1)	KT163250: 1589/1599 (99.4 %)
March 2011	703	0/40 (0)	-
April 2011	606	0/26 (0)	-
May 2011	555	2/29 (6.9)	KT163248: 1588/1599 (99.3 %)
			KT163257: 357/357 (100 %)
Total		5/184 (2.7)	

^a^Bat population numbers were counted, using the bounded count method [22], by 10 skilled forest staff

^b^Pooled bat urine sample collected under the trees

^c^AF376747: Nipah virus genome isolated from *Pteropus hypomelanus* in Malaysia

In addition, whole N gene sequences from six urine specimens of *P. lylei,* collected during 2010–2011 from central Thailand (see the map in Fig. 1), were included in the study. The sample collection and amplification methods were the same as for *P. hypomelanus*.”

## Results

Between 24 and 40 pooled bat urine samples were collected each trip (Table 1). NiV RNA was found in 5/184 (2.7 %) samples; in April 2010 (2 of 28, 7.1 %), February 2011 (1 of 24, 4.1 %) and May 2011 (2 of 29, 6.9 %) (Table 1). All NiV sequences (357 bp) shared 98.6–100 % identity with NiV-MY from the Malaysian *P. hypomelanus* (AF376747) [7]. Four of 5 PCR positive specimens were successfully sequenced further for the complete coding domains of the N gene (1599 bp). They showed 99.1–99.4 % identity to NiV-MY from *P. hypomelanus* (Table 1). Whole N gene sequencing of sample no. 18254 (KT163257) with 100 % identity to Malaysian *P. hypomelanus* NiV (357 bp) was not achieved due to low amount of viral RNA.

Phylogenetic analyses using maximum-likelihood methods from 357 and 1599 bp of N gene are shown in Fig. 1a and b respectively. Sequences were analyzed with available NiV sequences from bats, humans and pigs in GenBank, and six additional whole N gene sequences of NiV-BD obtained in 2010–2011 from urine of *P. lylei* bats in central Thailand. The NiVs from *P. hypomelanus* in this study form a monophyletic clade with other NiV-MY (Fig. 1a and b). All of NiV-MY (partial N gene, Fig. 2a) from *P. lylei* clustered with *P. hypomelanus* NiVs from southern Thailand (this study) and Malaysia.

Further, the similarity between the whole NiV-BD N gene from Thai *P. lylei* (KT163251- KT163256) and Bangladeshi patient (AY988601) was 99.1–99.2 % (Fig. 2b). The nucleotide identity between NiV-N gene (205 bp) from *P. giganteus* in India [14] (JF899339, not included in the phylogenetic tree) and NiVs found in Thailand from *P. hypomelanus* (no.s 1353, 1753, 8554, 16454) and *P. lylei* (no.s 1054, 3054, 15053, 15553, 14653, 33654) were 97.1 and 99.5 % respectively.

**Fig. 3:**
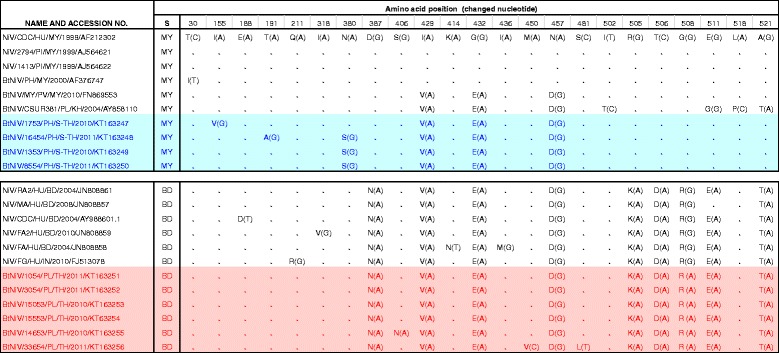
Amino acid (aa) differences among available complete NiV N gene open reading frame sequences (ORF). A total of 533 aa sequences were compared among NiV isolates from *P. hypomelanus* (blue) and *P. lylei* (red) from Thailand and NiV aa sequences available in GenBank. “.” Dots indicate sequences identical to AF212302; S, strain classification; MY, sequences from NiV-MY (Malaysia); BD, sequences from NiV-BD (Bangladesh); Bt, Bat; HU, Human; PI, Pig; PV, *Pteropus vampyrus*; PH, *P. hypomelanus*; PL, *P. lylei*; MY, Malaysia, BD, Bangladesh; IN, India; KH, Cambodia; TH, Thailand; S-TH, Southern Thailand

The visual alignment of the amino acid (aa) sequences was constructed from 533 aa of 1599 nucleotides of N gene open reading frame sequences (Fig. 3). The aa difference of N gene between strains NiV-MY and -BD sequences were found at 5 domains (positions 387, 505, 506, 508 and 521). The N-aa sequences between sick pigs and patients from Malaysia were identical, while one aa sequence difference was observed in NiV isolate from *P. hypomelanus* in Malaysia (Fig. 3). There were 5–6 aa sequence differences between full-length N in NiV isolates from *P. hypomelanus* from southern Thailand and *P. hypomelanus* in Malaysia. However, they had only 1–2 aa sequence differences when compared with NiV isolate from *P. vampyrus* in Malaysia, and 4–5 aa sequence differences with NiV from *P. lylei* in Cambodia. Interestingly, N-aa sequences among four NiV-BD isolates from *P. lylei* in central Thailand (GenBank accession nos. KT163251- KT163254) and from Bangladeshi patient (GenBank accession no. JN808861) were identical (Fig. 3).

## Discussion and conclusion

Whether *P. hypomelanus* or *P. vampyrus* bats were the spill-over hosts in the Malaysian outbreak has been unclear. Evidence in support of *P. hypomelanus* as the spill-over host is based on the near identical NiV sequence from this species to the other three NiVs isolated from human patients, and it was isolated shortly after the Malaysian outbreak [7]. In addition, the amino acid sequences obtained from NiV’s whole genome differed from NiV from two infected pigs at merely 3 positions [20]. Nonetheless, NiV sequences from *P. vampyrus* bats have more differing nucleotides to the isolates from the patients during the outbreak than NiV from *P. hypomelanus*, 98 and 6 nucleotides, respectively.

Our study at the southern island confirmed the similarity between NiV-MY from *P. hypomelanus* from Thailand and the Malaysian outbreak (in humans and pigs), and was in accord with the finding from one *P. hypomelanus* from Tioman island, Malaysia, about 960 km from our surveyed site [7]. From our finding, it remains possible that *P. hypomelanus* is restricted to NiV-MY, as only this strain was found in the 5 pooled urine specimens positive for NiV. Unfortunately, there is limited data to support host specificity as only one NiV-MY from *P. hypomelanus* has been reported from Malaysia [7]. A comprehensive analysis of bats across their migratory routes in Indo-Australian region should be conducted to clearly understand the ecology and transmission of NiV in *P. hypomelanus*.

The co-circulation of NiV-BD and NiV-MY strains within population of *P. lylei* in central Thailand [15] remains a mystery. High percent similarity of N nucleotide sequences from *P. lylei* and *P. giganteus*, and NiV patients in Bangladesh and the identity of whole N-aa among NiV isolates from *P. lylei* in Thailand and NiV patients in Bangladesh (Fig. 3), extrapolates the possibility of *P. lylei* bat in Thailand as a source of NiV infection to humans or other animals. The phylogenetic analysis on 357 bp (Fig. 2a) revealed that NiV-MY in *P. lylei* from Thailand, and *P. hypomelanus* from Malaysia and Thailand clustered together. This finding introduces the possibility of NiV transmission between *P. lylei* and *P. hypomelanus*, in addition to the previously suggested transmission between *P. vampyrus* and *P. lylei* [11]. Furthermore, the higher percent similarity of NiV N-aa between *P. hypomelanus* in Thailand and *P. vampyrus* in Malaysia than *P. hypomelanus* in Malaysia, raise the possibility of transmission between *P. hypomelanus* and *P. vampyrus*. Molecular characterization of NiV N and other genes, particularly those encoding the envelop glycoprotein from *P. vampyrus* in Thailand is required to investigate this possibility.

The alarming issue is that many bat species can harbor NiV. NiV-BD has been found in *P. giganteus* (India) [14], and *P. lylei* (Thailand) [16]. NiV-MY has been found in several bats species in many countries; *P. hypomelanus* (Malaysia [7] and Thailand [this study]), *P. vampyrus* (Malaysia [11] and Indonesia [13]), *P. lylei* (Cambodia [12] and Thailand [15]), *Hipposideros larvatus* (Thailand) [16], *Taphozous* species (Thailand) [15] and *Rousettus amplexicaudatus* (East Timor) [21]. The wide susceptibility of NiV to multiple host species allows for expedition of a larger-scale outbreak.

In conclusion, detecting NiV-MY strain in *P. hypomelanus* in southern Thailand, whose sequences share similarities to that identified in the Malaysian outbreak, and to *P. lylei* bats in central Thailand, suggests a potential role of *P. hypomelanus* as a possible source for inter-species transmission. This may result in the emergence of NiV infection to other animals and humans in both southern and central Thailand. Continuing surveillance of NiV infection in bats, humans, and pigs is vital for early detection and can potentially reduce the scale of an outbreak.

### Ethics approval and consent to participate

Bat urine samples were collected with permission from the Department of National Parks, Wildlife and Plant Conservation (No. 0907.1/20713). The urine specimens were collected under roost trees, no bat was caught for sample collection.

### Consent for publication

Not applicable.

### Availability of data and materials

The GenBank accession numbers for Nipah virus sequences reported in this paper are: KT163247- KT163250 for whole N gene and KT163257 for 357 bp partial N gene from *Pteropus hypomelanus* and KT163251- KT163256 for whole N gene from *P. lylei*.

## Abbreviations

BD: Bangladesh
bp: base pairs
IgG: immunoglobulin G
MY: Malaysia
N fragment/gene: nucleocapsid fragment/gene
NiV: Nipah virus
NiV-BD: NiV Bangladesh strain [NiV strain from the Bangladeshi and Indian outbreaks]
NiV-MY: NiV Malaysia strain [NiV strain from the Malaysian outbreak]
nt: nucleotides
PCR: Polymerase Chain Reaction
RNA: ribonucleic acid
RT-PCR: Reverse Transcription - Polymerase Chain Reaction
